# Morphine-induced hepatotoxicity in rats: Sex and dose variations in liver enzymes, oxidative stress, inflammation, apoptosis, and histopathology

**DOI:** 10.17305/bb.2026.13663

**Published:** 2026-05-28

**Authors:** Olaoluwa Sesan Olukiran, Oluwole Ojo Alese, Oluwadare Joshua Ogundipe, Stephen Taiye Adelodun, Rufus Ojo Akomolafe

**Affiliations:** 1 Department of Physiological Sciences, Faculty of Basic Medical Sciences, Obafemi Awolowo University, Ile Ife, Nigeria; 2 Institute of Neurological Disease, Gladstone Research Institutes, San Francisco, California, USA; 3 CentraCare Research Institutes, University of Minnesota Medical School, CentraCare Regional Campus, Saint Cloud, Minnesota, USA; 4 Department of Human Physiology, Osun State University, Osun State, Nigeria; 5 Department of Anatomy, Babcock University, Ilisan-Remo, Ogun State, Nigeria

**Keywords:** Liver enzymes, hepatocytes, sexual dimorphism, oxidative stress, rats

## Abstract

Drug-induced liver injury is a significant cause of acute liver failure, and sex-related biological differences may affect hepatic responses to xenobiotics. This study examined the sex- and dose-dependent effects of morphine on liver function, oxidative stress, inflammation, apoptosis, and histology in Wistar rats. Forty rats (20 males and 20 females) were administered distilled water or morphine at doses of 20, 40, or 60 mg/kg orally once daily for 30 days. Plasma liver enzyme levels and markers of hepatic oxidative stress, inflammation, and apoptosis were assessed, and liver tissues were analyzed histologically. Morphine significantly elevated alanine aminotransferase (ALT) activity in both sexes at most dose levels, with female rats exhibiting greater increases than males at 20 and 40 mg/kg. Alterations in aspartate aminotransferase (AST), alkaline phosphatase (ALP), and gamma-glutamyl transferase (GGT) were most pronounced in the low-dose groups. Low-dose morphine also led to significant increases in tumor necrosis factor-alpha (TNF-α), caspase-3, malondialdehyde (MDA), and nitric oxide (NO), indicating enhanced inflammatory, pro-apoptotic, and oxidative responses. While superoxide dismutase (SOD) and catalase (CAT) levels were not significantly altered, glutathione (GSH) demonstrated sex-related variation. Histological analysis revealed that male livers were largely preserved, whereas female livers displayed widened sinusoids and hepatocellular erosion. Overall, morphine induced sex- and dose-dependent hepatotoxicity, with females exhibiting greater susceptibility, particularly at low-dose exposure, which resulted in the most pronounced biochemical disturbances.

## Introduction

Drug-induced liver injury (DILI) is a significant concern in clinical practice, often resulting in the withdrawal of medications from the market. In Western countries, DILI is the leading cause of acute liver failure, with a considerable proportion of affected patients ultimately requiring liver transplantation [1]. Annually, an estimated 14–19 individuals per 100,000 are impacted, with approximately one-third of these cases being severe enough to cause jaundice [2, 3]. The differences in liver damage between males and females are influenced by various factors, primarily hormonal variations in females. Women are approximately 1.5 times more likely than men to develop an adverse drug reaction (ADR) [4]. This increased susceptibility is largely attributed to sex-related biological differences and a slower metabolic capacity, rendering women more vulnerable to drug interference events [5]. These differences encompass variations in pharmacokinetics and pharmacodynamics, alongside distinct patterns in immune response and inflammation. Specifically, females often exhibit a more pronounced inflammatory profile, characterized by elevated levels of hepatic pro-inflammatory cytokines, increased antibody production, and a greater predisposition to severe hepatitis. Furthermore, sex hormones appear to play a modulatory role in liver health [6]. Evidence from animal models indicates that estrogens may mitigate liver injury, whereas progesterone may exacerbate it, likely through their effects on inflammation and immune activity [7]. Additionally, Bizzaro *et al*. [8] demonstrated that in mouse models of acute liver injury, males experienced delayed recovery compared to females, attributing this sex-dependent difference to a greater influx of monocytes expressing the androgen receptor (AR) in male animals.

Opioids serve as key medications for pain management [9]. In recent years, opioid abuse has surged, primarily due to increased access to prescription opioids [10]. Morphine, a commonly used opioid, is effective in managing severe pain and is predominantly metabolized in the liver [11]. Experimental studies have shown that chronic morphine administration can induce hepatotoxicity [12]. Free radicals, generated during cellular metabolic processes, are implicated in hepatotoxicity [13], known to cause oxidative damage to lipids, proteins, and nucleic acids [14].

The liver plays a vital role in metabolism and detoxification, serving as the body’s primary defense against toxins transported from the intestine via the portal vein. Despite its essential functions, the liver can be compromised by various factors, including aging, physical trauma, and disease [15]. Hepatic function may also exhibit sex-dependent characteristics. The female hormone 17β-estradiol enhances the sensitivity of hepatic Kupffer cells to endotoxin in rats [16], suggesting that female livers may be more efficient in neutralizing substances carried through the portal vein. Notably, steroid hydroxylation patterns differ between sexes, with numerous cytochrome P450 (CYP450) superfamily members expressed in a sex-dependent manner in the liver [17]. Gene expression in male and female livers exhibits significant sexual dimorphism [18]. Male rats typically show a higher liver-to-body-weight ratio, with enhanced alcohol clearance and lipid metabolism compared to females [19, 20]. Conversely, females demonstrate superior cholesterol metabolism [21, 22]. A proteomic study indicated that sex has a more profound impact on liver protein expression than nutritional status [23]. Animal studies have revealed considerable sex-dependent differences in liver responses between male and female rats.

It is essential to consider sex differences in the impact of drug use and abuse on vital organs. However, there is limited literature regarding sex differences in the effects of morphine on the livers of male and female rats. Understanding this sexual dimorphism is crucial for elucidating variations in liver function and response. Therefore, this study aims to investigate the sexual dimorphism in the effects of morphine administration on the livers of male and female Wistar rats.

## Materials and methods

### Animal management

The experiment was conducted in accordance with the Animal Welfare Act and the Guidelines for Animal Experimentation established by the Osun State University Animal Health and Ethics Research Committee, with ethical approval number UNIOSUNHREC 2025/004C. This study utilized 40 Wistar rats, evenly divided into 20 males and 20 females, each weighing between 120 and 150 grams at the study’s outset. The rats were procured from the Animal Holdings of the College of Health Sciences, Osun State University, Osogbo, Osun State, Nigeria, where the study was also conducted. They were housed in polycarbonate cages under a natural light/dark cycle for two weeks prior to the experiment, with unrestricted access to standard rat pellets and water.

### Experimental protocol

The rats were assigned to four experimental groups, maintaining an equal sexual distribution of 5 males and 5 females in each group. Control animals received distilled water orally at a dose of 0.5 mL per 100 g of body weight. In contrast, the experimental groups were administered daily oral doses of morphine at 20, 40, or 60 mg/kg for 30 days. Following this treatment period, the rats were anesthetized with ketamine (10 mg/kg) and underwent cardiac puncture to obtain blood samples, which were collected in bottles containing lithium heparin. Plasma for liver function marker assays was obtained by centrifuging the blood samples in a cold centrifuge (Centurium Scientific Model, 8881) at 3000 rpm for 10 min. Subsequently, the livers were carefully excised and weighed. For biochemical analysis, 2.5 g of liver tissue was homogenized using an Electric Homogenizer (SI601001) in 10 mL of 0.25 M sucrose solution. The resulting homogenate was subjected to centrifugation at 3000 rpm for 20 min, and the supernatant was utilized to estimate markers of antioxidant enzymes, inflammation, and apoptosis. Remaining liver tissue was fixed in 10% formo-saline for histological analysis.

### Assessment of aspartate aminotransferase (AST), alanine aminotransferase (ALT), alkaline phosphatase (ALP), and gamma-glutamyl transferase (GGT)

Plasma activities of AST, ALT, ALP, and GGT were determined using commercial colorimetric kits (Randox Laboratories, Crumlin, Antrim, UK) following the manufacturer’s protocols.

### Evaluation of biomarkers of oxidative stress and lipid peroxidation

#### Estimation of malondialdehyde (MDA)

Lipid peroxidation was quantified by measuring MDA levels according to the thiobarbituric acid reactive substances (TBARS) method as described by [24]. For deproteinization, 200 µL of the standard or supernatant was mixed with 500 µL of trichloroacetic acid (TCA) and centrifuged at 3000 rpm for 10 min. Subsequently, 1 mL of 0.75% thiobarbituric acid (TBA) was added to 0.1 mL of the supernatant. This mixture was incubated in a hot water bath at 100 ^∘^C for 20 min and then cooled immediately in ice water. Sample and standard absorbances were measured at 532 nm against a reagent blank using a spectrophotometer.

#### Determination of glutathione

Glutathione levels in liver homogenates were determined following the protocol of Tietze [25]. Glutathione was oxidized to form a yellow 5′-thio-2-nitrobenzoic acid using 5,5′-dithio-bis-(2-nitrobenzoic acid), which serves as an oxidizing sulfhydryl reagent. To 500 µL of liver tissue supernatant, 4% sulfosalicylic acid was added to precipitate the glutathione. This mixture was incubated at 4^∘^C for one hour, followed by centrifugation at 1200 g for 20 min to obtain a clear supernatant. Approximately 33 µL of the supernatant was mixed with 900 µL of potassium phosphate buffer (0.1 M, pH 7.4) and 66 µL of 100 mM 5,5′-dithio-bis-(2-nitrobenzoic acid). The resulting yellow-colored complex was quantified spectrophotometrically at an absorbance of 412 nm.

#### Determination of superoxide dismutase

Superoxide dismutase (SOD) activity was measured as previously described [26]. To 0.2 mL of the diluted sample, 2.5 mL of 0.05 M carbonate buffer (pH 10.2) was added for equilibration in the spectrophotometer. The reaction was initiated by the addition of 0.3 mL of freshly prepared 0.3 mM adrenaline to the mixture, which was then thoroughly mixed by inversion. The reference cuvette contained 2.5 mL of buffer, 0.3 mL of substrate (adrenaline), and 0.2 mL of water. Absorbance at 480 nm was monitored at 30-second intervals over a period of 150 s.

#### Estimation of catalase (CAT)

CAT activity was assessed using a modified method from [27]. In a cuvette, 450 µL of a 50 mM phosphate buffer (pH 7.4) was combined with 2000 µL of 10 mM H_2_O_2_ and 1000 µL of the sample supernatant. Absorbance was recorded at 240 nm for 1 minute using a spectrophotometer. The molar extinction coefficient of H_2_O_2_, 0.0436 mM^−1^cm^−1^, was utilized to calculate CAT activity. One unit of CAT activity is defined as the amount of enzyme that degrades 1 mmol of H_2_O_2_ per minute, expressed as units/mg.

#### Determination of nitric oxide

Nitric oxide levels in liver tissue were determined spectrophotometrically by incubating a 300 µL sample with 100 µL of Griess reagent for 30 min at room temperature. A blank, consisting of 100 µL of Griess reagent and 2.9 mL of distilled water, was prepared for calibration.

### Assessment of markers of inflammation and apoptosis

The concentrations of tumor necrosis factor-alpha (TNF-α) and caspase-3 were measured using a sandwich enzyme-linked immunosorbent assay (ELISA) kit from Nanjing Mornmed Medical Equipment Co., Ltd., Jiangsu Province, China. The intra-assay coefficient of variation (CV) was less than 10%, while the inter-assay CV was below 15%. The assay range and sensitivity were 20 pg/mL – 640 pg/mL and < 1.0 pg/mL for TNF-α, and 1.5 pmol/L – 48 pmol/L and < 0.1 pmol/L for caspase-3, respectively. Briefly, 10 µL of each sample was mixed with 40 µL of sample diluent in the sample wells, while 50 µL of TNF-α and caspase-3 standards were added to the standard wells. Following preparation, 100 µL of horseradish peroxidase (HRP)-conjugate reagent was added to all wells except the blank, and the plate was incubated for 60 min at 37^∘^C. To remove unbound antigens, the wells were washed five times with washing buffer, incorporating a 30-second soak period before each drainage. The colorimetric reaction was initiated by adding 50 µL each of Chromogen Solutions A and B, followed by a 15-minute incubation at 37^∘^C. The reaction was then halted using 50 µL of stop solution. Within 15 min of termination, optical density was measured spectrophotometrically at 450 nm against the blank and standards. Final TNF-α and caspase-3 concentrations were determined by comparison with standard curves and expressed in pg/mL and ng/mL, respectively.

### Histopathological assessment

Following fixation, liver tissues were processed for histology by dehydration through a graded alcohol series, followed by clearing in xylene and embedding in paraffin wax. Sections 5–6 µm thick were prepared using a microtome, mounted on slides, and stained with hematoxylin-eosin (H&E). The slides were examined using an Olympus CH light microscope (Olympus, Tokyo, Japan). Photomicrographs were captured at 400× magnification using a Leica DM 750 camera.

### Statistical analysis

Data are presented as the mean ± standard error of the mean (SEM). Statistical comparisons between experimental groups and the control were conducted using one-way analysis of variance (ANOVA) followed by the Newman–Keuls post-hoc test. Sexual comparisons (male vs. female) were analyzed using an unpaired two-tailed Student’s *t*-test. Statistical computations were performed using GraphPad Prism version 5.03 (GraphPad Software, La Jolla, CA, USA), with *P* < 0.05 indicating statistical significance.

### Ethical statement

The animal study protocols for this research were reviewed and approved by the Health Research Ethics Committee, College of Health Sciences, Osun State University, Osogbo, Osun State, Nigeria (Approval No. UNIOSUNHREC 2025/004C). This approval was granted in accordance with the EU Directive 2010/63/EU.

## Results

### Sex and dose differences in AST, ALT, ALP and GGT activities of rats administered with different doses of morphine

Plasma AST activity in male rats showed no significant difference between the morphine-treated and control groups (*F* ═ 0.419; *P* ═ 0.162). In contrast, female rats administered 20 mg/kg morphine exhibited significantly elevated plasma AST levels compared to both the control group and other morphine-treated groups (*F* ═ 152.9; *P* < 0.0001). Male rats given 20 and 40 mg/kg morphine displayed significantly lower plasma AST activity compared to their female counterparts (*t* ═ 13.60; *P* < 0.0001 for 20 mg/kg; *t* ═ 11.33; *P* < 0.0001 for 40 mg/kg) (Figure 1A).

**Figure 1.**
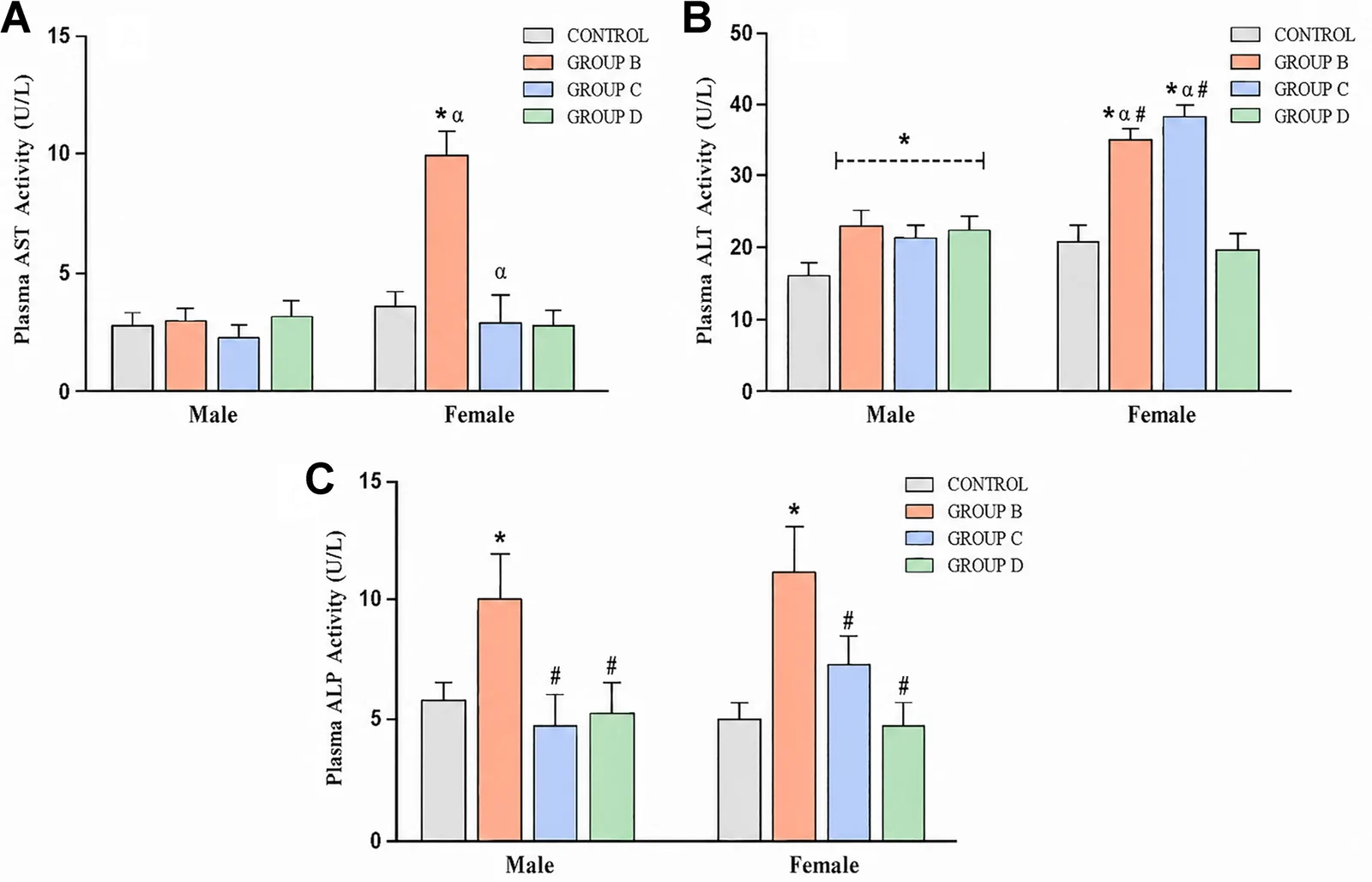
**Sex- and dose-dependent changes in plasma AST, ALT, and ALP activities in Wistar rats after 30 days of oral morphine administration.** (A) Plasma AST activity. AST did not change significantly in male rats, whereas female rats treated with 20 mg/kg morphine showed a marked increase compared with the female control group and the other female morphine-treated groups; females also exhibited higher AST activity than males at 20 and 40 mg/kg. (B) Plasma ALT activity. ALT was significantly increased in all morphine-treated male groups and in female rats treated with 20 and 40 mg/kg; female rats had higher ALT activity than males at 20 and 40 mg/kg, while the female 60 mg/kg group showed lower ALT activity than the female 20 and 40 mg/kg groups. (C) Plasma ALP activity. ALP activity was significantly elevated at 20 mg/kg in both sexes compared with the respective control group and the other morphine-treated groups. Data are presented as mean ± SEM (*n* ═ 5 per sex per group). Control rats received distilled water; Group B received morphine 20 mg/kg; Group C received morphine 40 mg/kg; and Group D received morphine 60 mg/kg. *P < 0.05 vs the respective control group; α*P* < 0.05 vs males at the same dose; #*P* < 0.05 vs Group B within the same sex. Within-sex comparisons were performed using one-way analysis of variance followed by Newman–Keuls post hoc test, whereas male-versus-female comparisons at the same dose were performed using an unpaired two-tailed Student’s *t*-test. **Abbreviations:** ALP, alkaline phosphatase; ALT, alanine aminotransferase; AST, aspartate aminotransferase; SEM, standard error of the mean.

Both male and female rats exhibited significantly elevated ALT activities (*F* ═ 27.43; *P* < 0.0001 and *F* ═ 168.9; *P* < 0.0001, respectively) in the groups treated with the three doses of the drug compared to their respective control groups, except for the female rats treated with 60 mg/kg of morphine. Female rats receiving 60 mg/kg morphine showed significantly lower ALT activity than those treated with 20 and 40 mg/kg morphine. Additionally, male rats treated with 20 and 40 mg/kg morphine exhibited significantly lower ALT activity than their female counterparts, with highly significant differences: (*t* ═ 12.59; *P* < 0.0001) for the 20 mg/kg group and (*t* ═ 13.78; *P* < 0.0001) for the 40 mg/kg group (Figure 1B).

Plasma ALP activities were significantly elevated in both male and female rats given 20 mg/kg morphine (*F* ═ 6.537; *P* ═ 0.004 and *F* ═ 3.699; *P* ═ 0.034, respectively). This increase was observed when compared to control rats and those receiving other morphine doses across both sexes. Male rats exhibited significantly reduced ALP activity compared to female rats across all three morphine doses used in the study (*t* ═ 0.354; *P* ═ 0.366; *t* ═ 1.134; *P* ═ 0.145; and *t* ═ 0.216; *P* ═ 0.417, respectively) (Figure 1C).

A significant increase in plasma GGT activity was observed in male rats treated with the low dose of morphine (*F* ═ 6.443; *P* ═ 0.005) compared to the control group and those receiving medium or high doses. Female rats administered 20 mg/kg morphine exhibited significantly elevated plasma GGT activity (*F* ═ 5.518; *P* ═ 0.029) compared to the control group. Conversely, GGT activity in female rats treated with medium and high doses of the drug was markedly reduced compared to those given the low dose. Both male and female rats receiving medium and high doses of morphine demonstrated a substantial increase in plasma GGT activity compared to the control group. However, no significant differences in GGT activity were noted between male and female rats across all three morphine doses (*t* ═ 1.821; *P* ═ 0.053; *t* ═ 0.*178; P* ═ 0.432; and *t* ═ 0.82; *P* ═ 0.216, respectively) (Figure 2A).

**Figure 2.**
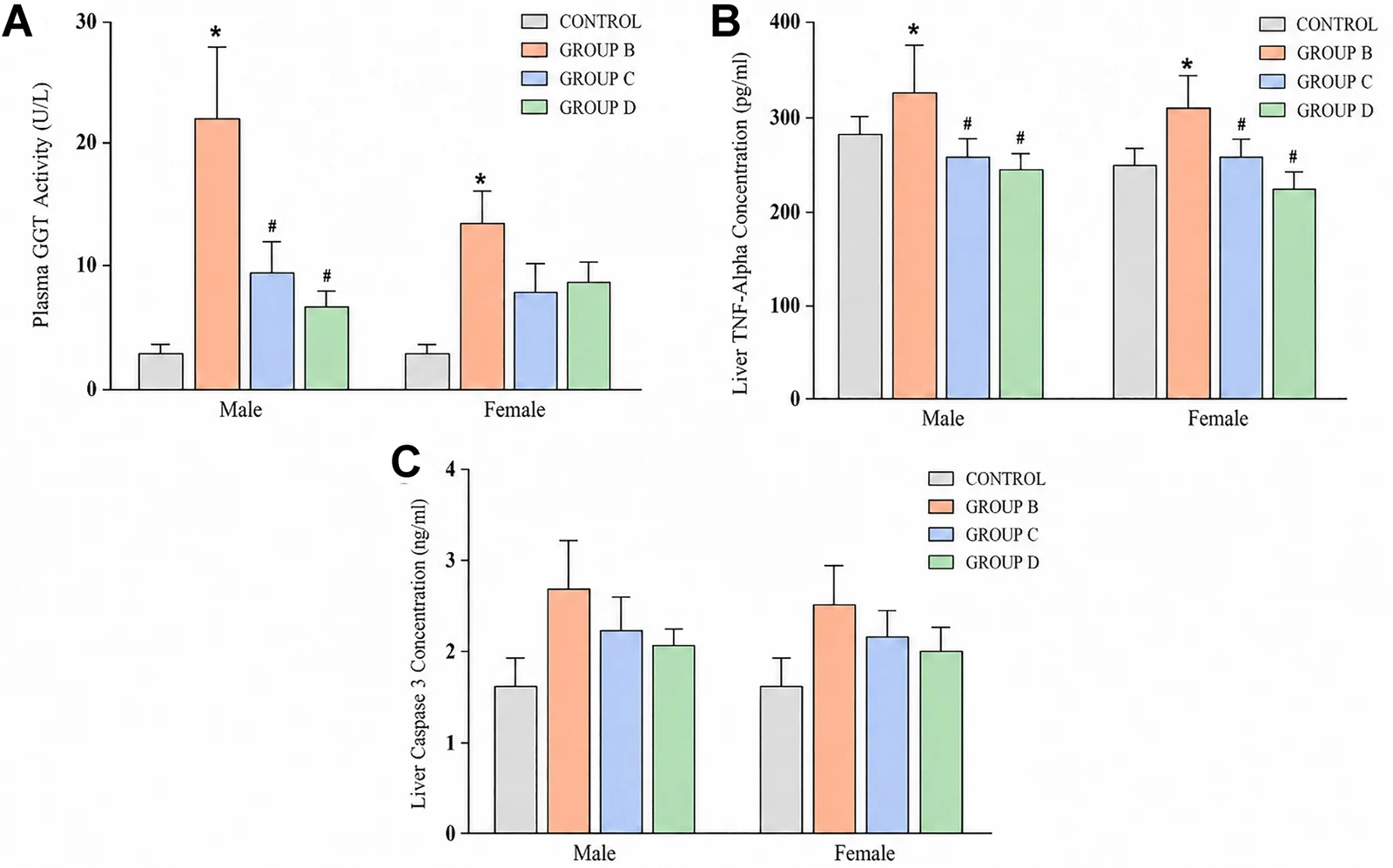
**Sex- and dose-dependent effects of repeated oral morphine administration on plasma gamma-glutamyl transferase activity and hepatic inflammatory and apoptotic markers in Wistar rats.** (A) Plasma GGT activity. In male rats, GGT activity was significantly increased in the 20 mg/kg morphine group compared with the control and the 40 and 60 mg/kg groups. In female rats, the 20 mg/kg group also showed significantly elevated GGT activity compared with the control group, whereas the 40 and 60 mg/kg groups were lower than the 20 mg/kg group. No significant sex-related differences in GGT activity were observed at any dose level. (B) Hepatic TNF-α concentration. TNF-α levels were significantly increased in the 20 mg/kg morphine group in both sexes compared with the respective control group and the higher-dose groups, while no significant male-versus-female differences were detected at any dose. (C) Hepatic caspase-3 concentration. Caspase-3 concentrations were highest in the 20 mg/kg morphine group in both sexes and lower in the 40 and 60 mg/kg groups; however, neither within-sex comparisons versus controls nor between-sex comparisons reached statistical significance. Data are presented as mean ± SEM (*n* ═ 5 per sex per group). Control rats received distilled water; Group B received morphine 20 mg/kg; Group C received morphine 40 mg/kg; and Group D received morphine 60 mg/kg. ^*^P < 0.05 vs the respective control group; #*P* < 0.05 vs Group B within the same sex. Within-sex comparisons were performed using one-way analysis of variance followed by Newman–Keuls post hoc test, whereas male-versus-female comparisons at the same dose were performed using an unpaired two-tailed Student’s *t*-test. **Abbreviations:** GGT, gamma-glutamyl transferase; SEM, standard error of the mean; TNF-α, tumor necrosis factor-alpha.

### Sex and dose differences in TNF-α and caspase-3 levels of rats treated with different doses of morphine

In male rats, TNF-α levels significantly increased after treatment with the low dose of morphine compared to both the control group and rats receiving medium and high doses of the drug (*F* ═ 3792; *P* ═ 0.0001). Conversely, female rats treated with the low dose of morphine showed significantly higher TNF-α levels (*F* ═ 5.094; *P* ═ 0.012) compared to the control group and those treated with 40 and 60 mg/kg of morphine. No significant differences were observed in TNF-α levels of rats of either sex treated with medium and high doses of morphine compared to the control group. Furthermore, TNF-α levels in male rats across all three morphine doses did not significantly differ from those in female rats (*t* ═ 0.342; *P* ═ 0.371; *t* ═ 0.032; *P* ═ 0.488; and *t* ═ 1.237; *P* ═ 0.126, respectively) (Figure 2B).

Caspase-3 levels in both male and female control rats were not significantly different from those in rats treated with varying doses of morphine (*F* ═ 1.*850; P* ═ 0.179 and *F* ═ 1.409; *P* ═ 0.277, respectively). However, rats receiving the low dose of morphine exhibited considerably higher caspase-3 levels compared to those treated with medium and high doses. Caspase-3 levels in male rats treated with all three morphine doses showed no significant differences compared to those in female rats (*t* ═ 0.*237; P* ═ 0.409; *t* ═ 0.*490; P* ═ 0.319; and *t* ═ 0.*587; P* ═ 0.287, respectively) (Figure 2C).

### Sex and dose differences in malondialdehyde, reduced glutathione, superoxide dismutase, CAT and nitric oxide levels of rats treated with different doses of morphine

Male and female rats administered a low dose of morphine exhibited significantly elevated levels of MDA (*F* ═ 4.533, *P* ═ 0.018 for males; *F* ═ 7.563, *P* ═ 0.002 for females) compared to both the control group and those treated with medium and high doses of morphine. In contrast, rats receiving medium and high doses did not show significant differences in MDA levels relative to the control group. The MDA levels in male rats across all morphine doses were not significantly different from those of female rats (*t* ═ 0.346, *P* ═ 0.389; *t* ═ 0.437, *P* ═ 0.367; *t* ═ 0.051, *P* ═ 0.480) (Figure 3A).

**Figure 3.**
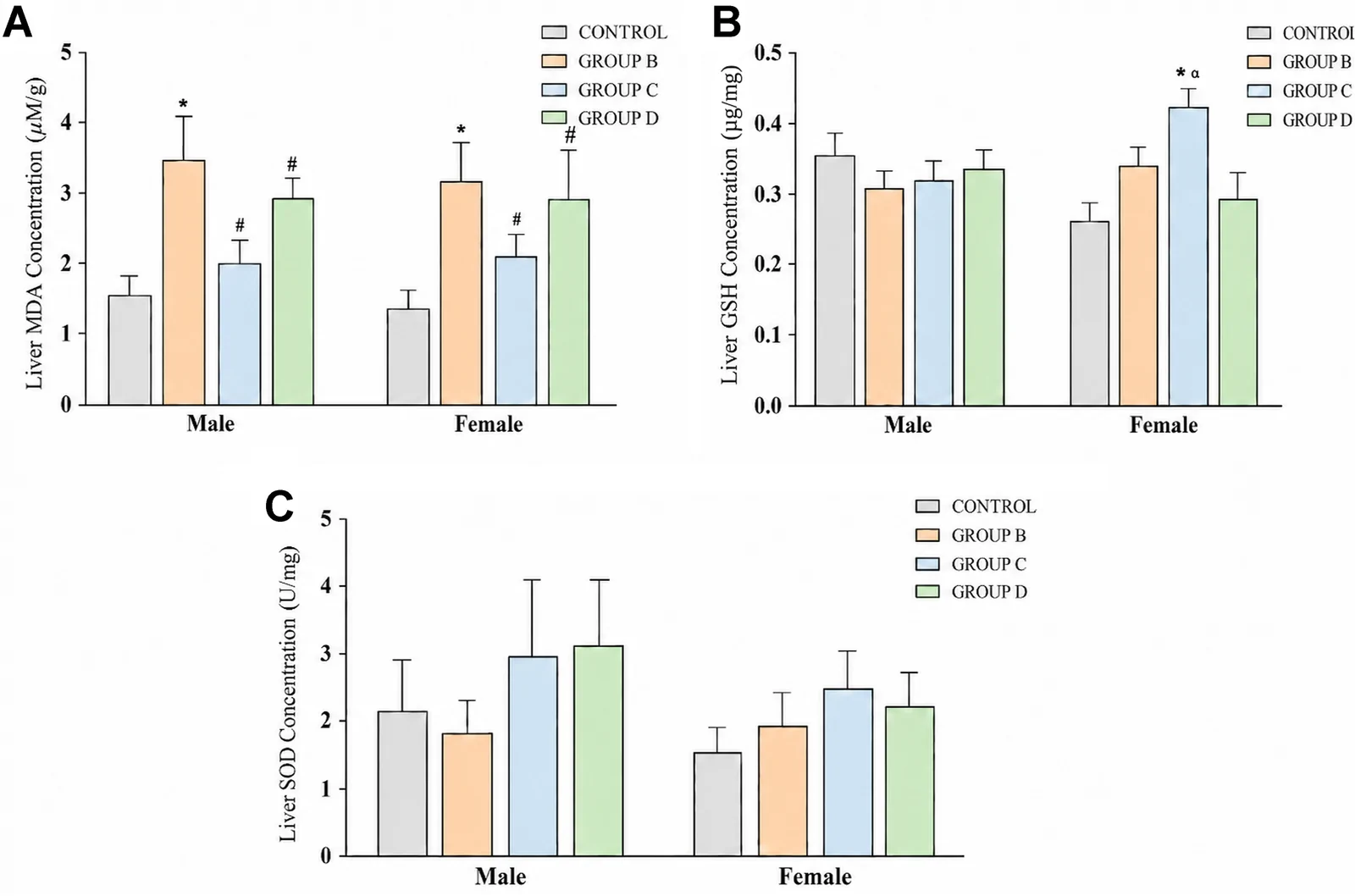
**Sex- and dose-dependent effects of repeated oral morphine administration on hepatic malondialdehyde, reduced glutathione, and superoxide dismutase in Wistar rats.** (A) Hepatic MDA concentration. MDA levels were significantly increased in the 20 mg/kg morphine group in both sexes compared with the respective control group and the 40 and 60 mg/kg groups, with no significant sex-related differences at any dose level. (B) Hepatic reduced GSH concentration. In male rats, GSH levels did not differ significantly from control values at any morphine dose. In female rats, GSH levels were higher in the 20 and 40 mg/kg groups, with a significant increase observed only at 40 mg/kg; at this dose, females also showed significantly higher GSH levels than males. (C) Hepatic SOD activity. SOD showed an upward trend in the 40 and 60 mg/kg groups in both sexes, but no significant differences versus controls or between sexes were detected. Data are presented as mean ± SEM (*n* ═ 5 per sex per group). Control rats received distilled water; Group B received morphine 20 mg/kg; Group C received morphine 40 mg/kg; and Group D received morphine 60 mg/kg. ^*^P < 0.05 vs the respective control group; α*P* < 0.05 vs males at the same dose; #*P* < 0.05 vs Group B within the same sex. Within-sex comparisons were performed using one-way analysis of variance followed by Newman–Keuls post hoc test, whereas male-versus-female comparisons at the same dose were performed using an unpaired two-tailed Student’s *t*-test. **Abbreviations:** GSH, reduced glutathione; MDA, malondialdehyde; SEM, standard error of the mean; SOD, superoxide dismutase.

The levels of reduced glutathione (GSH) in male rats treated with low, medium, and high doses of morphine were not significantly different from those in the control group (*F* ═ 0.411, *P* ═ 0.747). Conversely, GSH levels in female rats were significantly higher in those administered low and medium doses, with statistical significance achieved only in the medium dose group (*F* ═ 3.833, *P* ═ 0.0001). Male rats receiving low and high doses of morphine did not show significant differences in GSH levels compared to their female counterparts; however, male rats treated with the medium dose exhibited significantly lower GSH levels than females (*t* ═ 2.489, *P* ═ 0.019) (Figure 3B).

SOD activity in both male and female rats treated with all three morphine doses did not significantly differ from that of the control group (*F* ═ 0.441, *P* ═ 0.239). However, SOD activity was increased in both sexes for medium and high doses compared to the control and low dose groups. Across all three morphine doses, SOD activity in male rats was not significantly different from that in female rats (*t* ═ 0.036, *P* ═ 0.486; *t* ═ 0.343, *P* ═ 0.370; *t* ═ 0.701, *P* ═ 0.252) (Figure 3C).

CAT activity in both male and female rats treated with low, medium, and high doses of morphine did not significantly differ from their respective control groups (*F* ═ 0.580, *P* ═ 0.637 for males; *F* ═ 1.055, *P* ═ 0.397 for females). Nonetheless, the low dose group exhibited significantly lower CAT activity compared to both the control and other treated groups across sexes. Furthermore, CAT activity in male rats treated with all doses was not significantly different from that of female rats (*t* ═ 0.074, *P* ═ 0.471; *t* ═ 0.483, *P* ═ 0.321; *t* ═ 1.048, *P* ═ 0.163) (Figure 4A).

**Figure 4.**
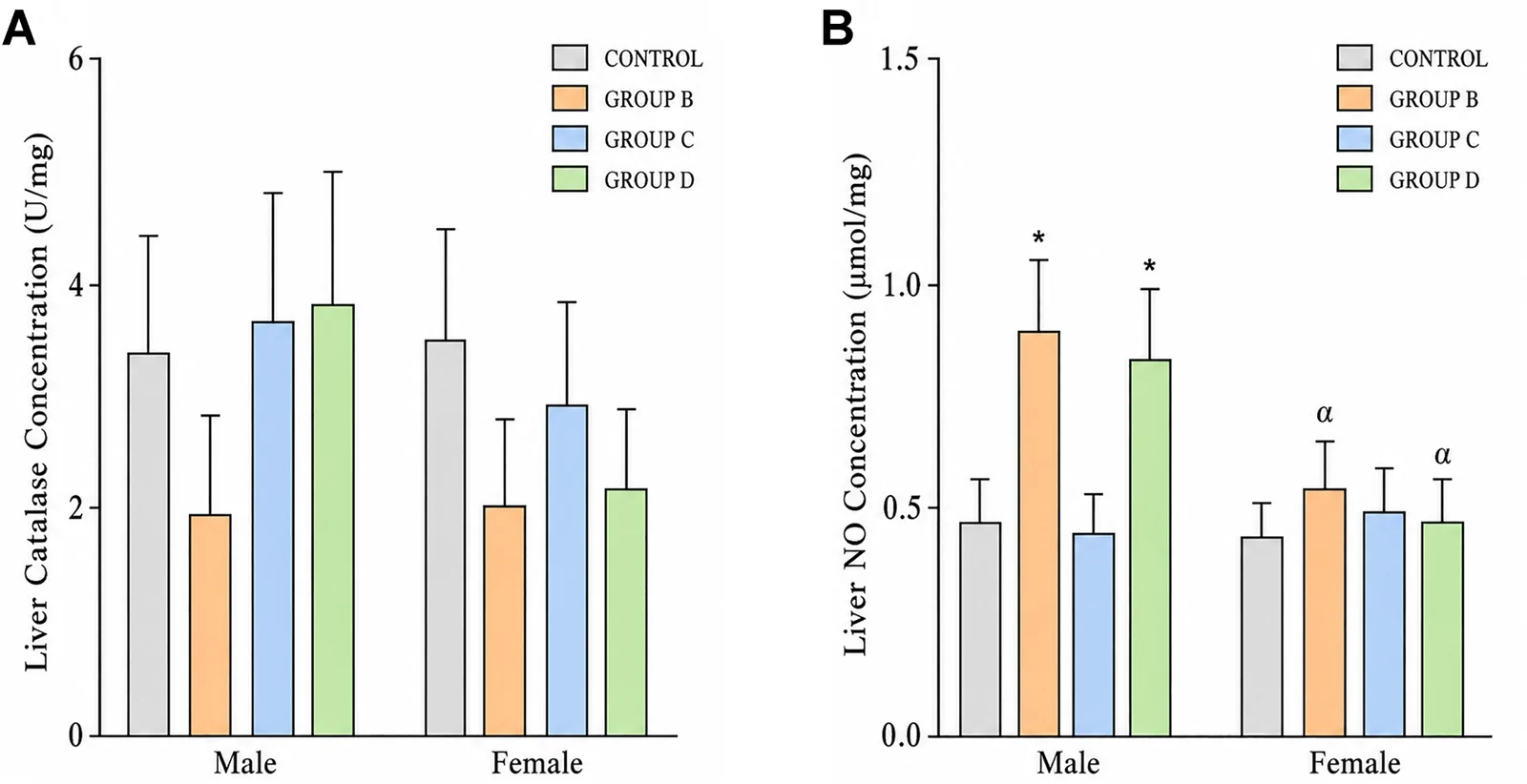
**Sex- and dose-dependent effects of repeated oral morphine administration on hepatic catalase activity and nitric oxide levels in Wistar rats.** (A) Hepatic CAT activity. CAT activity did not differ significantly from the respective control groups in either sex at any morphine dose. However, the 20 mg/kg morphine group showed lower CAT activity than the 40 and 60 mg/kg groups in both males and females, and no significant sex-related differences were detected at any dose. (B) Hepatic NO levels. In male rats, NO levels were significantly elevated in the 20 and 60 mg/kg morphine groups compared with the control group. In female rats, NO levels did not differ significantly from control values across the dose groups. Male rats treated with 20 and 60 mg/kg morphine exhibited significantly higher NO levels than females at the corresponding doses, whereas no significant sex difference was observed at 40 mg/kg. Data are presented as mean ± SEM (*n* ═ 5 per sex per group). Control rats received distilled water; Group B received morphine 20 mg/kg; Group C received morphine 40 mg/kg; and Group D received morphine 60 mg/kg. ^*^P < 0.05 vs the respective control group; α*P* < 0.05 vs males at the same dose. Within-sex comparisons were performed using one-way analysis of variance followed by Newman–Keuls post hoc test, whereas male-versus-female comparisons at the same dose were performed using an unpaired two-tailed Student’s *t*-test. **Abbreviations:** CAT, catalase; NO, nitric oxide; SEM, standard error of the mean.

Nitric oxide levels were significantly elevated in male rats treated with both low and high doses of morphine compared to the control group (*F* ═ 4.174, *P* ═ 0.023). In contrast, female rats showed no significant variation in nitric oxide levels across all dosage groups relative to controls (*F* ═ 1.046, *P* ═ 0.399). Male rats receiving low and high doses of morphine exhibited significantly higher nitric oxide levels than their female counterparts (low dose: *t* ═ 1.882, *P* ═ 0.048; high dose: *t* ═ 2.399, *P* ═ 0.022). However, no significant difference in nitric oxide levels was observed between male and female rats treated with the medium dose (Figure 4B).

### Photomicrographs of the liver of male rats treated with the three doses of morphine

The photomicrograph of the control group reveals normal hepatic histoarchitecture, with distinct hepatocytes and clear central veins. The experimental rats treated with 20, 40, and 60 mg/kg of morphine also displayed normal hepatocyte morphology and central veins without observable aberrations (Figure 5).

### Photomicrographs of the liver of female rats treated with the three doses of morphine

The photomicrograph of the control group shows normal liver histoarchitecture, characterized by well-defined hepatocytes and distinct central veins. Rats administered 20 mg/kg of morphine exhibited normal liver cell appearance with clear central veins and no observable distortion of liver histoarchitecture. However, rats treated with 40 and 60 mg/kg of morphine displayed erosion of liver tissue surrounding the hepatocytes due to necrosis and widened sinusoids compared to the control group (Figure 6).

**Figure 5.**
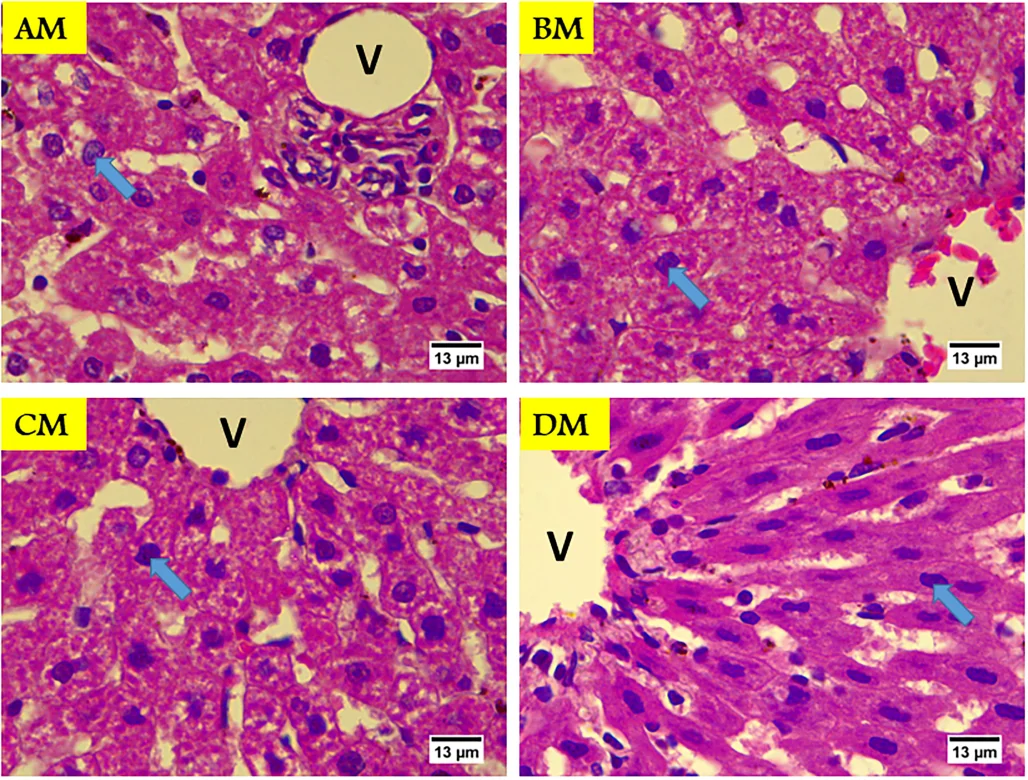
**Representative photomicrographs of liver sections from male Wistar rats following 30 days of oral morphine administration.** The control male group (AM) shows preserved hepatic architecture, with distinct hepatocytes (blue arrows) and a clearly defined central vein (V). Similarly, liver sections from males treated with morphine at 20 mg/kg (BM), 40 mg/kg (CM), and 60 mg/kg (DM) show preserved hepatocellular morphology and central venous architecture without evident histopathological abnormalities. Sections were stained with H&E; scale bar = 13 µm. **Abbreviation:** H&E, hematoxylin and eosin.

**Figure 6.**
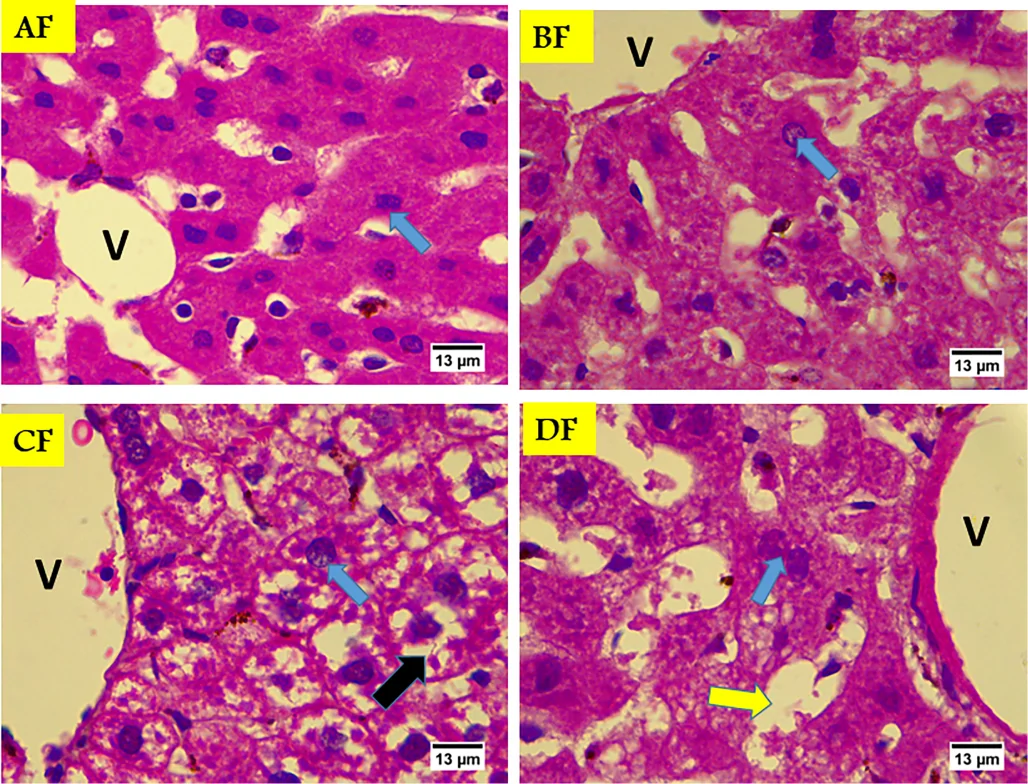
**Representative photomicrographs of liver sections from female Wistar rats following 30 days of oral morphine administration.** The control female group (AF) shows preserved hepatic histoarchitecture, with well-defined hepatocytes (blue arrow) and a clearly demarcated central vein (V). Similarly, liver sections from females treated with morphine at 20 mg/kg (BF) show preserved hepatocellular architecture without evident histopathological abnormalities. In contrast, sections from females treated with 40 mg/kg morphine (CF) demonstrate erosion of hepatic tissue around hepatocytes consistent with necrotic change (black arrow), whereas sections from females treated with 60 mg/kg morphine (DF) show widening of the hepatic sinusoids (yellow arrow). Sections were stained with H&E; scale bar = 13 µm. **Abbreviation:** H&E, hematoxylin and eosin.

## Discussion

Given that the liver is the principal site for morphine biotransformation, the drug can induce significant adverse physiological effects on this organ. This study investigated the impact of morphine on liver function, inflammation, and apoptosis markers in male and female rats, considering both sex and dose differences [28].

Morphine administration resulted in significant increases in ALT, AST, and ALP activities in both sexes compared to the control groups. Previous research has demonstrated that morphine can induce liver cell injury and genotoxicity in rat livers [29]. Damage or necrosis of hepatocyte membranes leads to the release of liver enzymes into the bloodstream [30]. Consequently, the elevated activities of hepatic biomarkers observed in this study indicate compromised hepatocellular function [31].

Sex has a critical role in drug-induced liver damage, as numerous studies consistently report that males are generally more susceptible to hepatotoxin-induced liver injury than females [32–34]. Drug bioactivation and the influence of estrogen are key factors explaining this sex-dependent susceptibility [33–35]. Variations in pharmacokinetics and pharmacodynamics between sexes characterize many drug treatments, elucidating individual differences in efficacy and toxicity.

Differences in drug metabolism largely account for sex-dependent variations in pharmacokinetics. Males and females exhibit different levels of hepatic enzymes essential for metabolizing drugs, steroids, fatty acids, and environmental chemicals, such as CYP450 enzymes, sulfotransferases, glutathione transferases, and uridine diphosphate (UDP)-glucuronosyl transferases. Similar to findings in neurons and splenocytes, hepatocytes and cholangiocytes demonstrate sex-linked variations in their toxicological responses and vulnerability [36, 37].

Conversely, some studies report that the risk of developing ADRs is approximately 1.5 times higher in females than in males [38]. Sex hormones contribute to sexual dimorphism, with estrogens generally reducing liver damage while progesterone may exacerbate it. This differential effect is likely mediated by distinct modulation of inflammation and immune responses, as evidenced in animal models [7]. Thus, the elevated liver enzyme activity observed in the plasma of female rats compared to males might be potentiated by female sex steroids, given that progesterone has been implicated in liver damage, despite numerous studies highlighting estrogen’s protective role in the liver.

In the differential expression of various CYP450 isoforms, it was noted that the CYP3A isoform displays greater activity in women than in men [5]. Conversely, expression levels of the drug efflux pump P-glycoprotein (P-GP), encoded by the multidrug resistance 1 (MDR1) gene, are higher in men, potentially explaining the lower toxicity rates observed in males. Male sex steroids negatively regulate P-GP expression, influencing drug absorption through the small intestine of rats [39]. CYP3A4 and CYP3A5, the most abundantly expressed drug-metabolizing enzymes in the human liver, are key members of the CYP450 superfamily, typically involved in the metabolism of 30%–50% of known drugs [40]. CYP3A4 is more highly expressed in women, closely linked to increased expression of the corresponding mRNA [41]. Additionally, elevated CYP2B6 activity has been observed in women, paralleling increased gene expression [42].

In this study, morphine significantly increased TNF-α levels in rats treated with a low dose of the drug, indicating a tissue response to cell injury, as described by Lamba *et al.* [43]. This response initiates with the release of pro-inflammatory markers such as IL-1 and TNF-α, which subsequently stimulate the production of various metabolites and cytokines, along with the release of arachidonic acid, prostaglandins (PGs), and leukotrienes [44]. The impact of morphine on inflammatory markers, particularly TNF-α, remains contentious in the literature. While some research, including Madera-Salcedo *et al.* [45], suggests morphine may reduce lipopolysaccharide-induced TNF-α release from mast cells, other studies, such as El-Hage *et al.* [46], indicate that it could exacerbate liver inflammation and elevate TNF-α levels in the context of human immunodeficiency virus type 1 (HIV-1) infection.

Differences in the severity of inflammatory diseases, such as atherosclerosis, neurological disorders, periodontitis, and rheumatoid arthritis, between sexes suggest that female sex hormones may modulate the inflammatory response [47]. A recent study underscored the protective role of estrogen receptors (ER) in the liver. All female and male mice administered estradiol (E2) survived liver ischemia and reperfusion injury. In stark contrast, all ERα-null male mice and 60% of ERα-null female mice succumbed within 3–5 days following the injury [48].

Similarly, studies in both human and animal models have demonstrated that females in the proestrus phase (characterized by elevated estrogen levels) tolerate trauma, hemorrhage, and sepsis significantly better than males [49]. This protective effect is, at least in part, attributed to the capacity of E2-activated ER to enhance heme oxygenase 1 (HO-1) expression, thereby mitigating the inflammatory response [50]. In response to systemic inflammation induced by endotoxin, female mice and males pretreated with E2 produced lower levels of TNF-α and macrophage inflammatory protein-1α compared to untreated male mice, conferring resistance to endotoxin-induced mortality [51]. The observed marginal downregulation of TNF-α in females relative to males in this study likely results from estrogen’s influence. The ER has the potential to modulate hepatic inflammation in a sex-dimorphic manner. Specifically, estrogen stimulation directs estrogen receptor alpha (ERα) to modulate the expression of pro-inflammatory cytokines in the mouse liver [52].

Apoptosis is primarily driven by members of the caspase family, activated by pro-apoptotic stimuli such as endoplasmic reticulum stress and reactive oxygen species (ROS). The present study revealed that morphine significantly upregulates caspase-3 expression in the livers of rats treated with a low dose of the drug, indicating a pro-apoptotic effect of morphine. Similarly, El-Sheikh and Azza [53] observed increased caspase-3 expression in the liver following morphine treatment, suggesting a consistent pro-apoptotic response in rats. However, this finding diverges from previous studies that reported morphine’s anti-apoptotic effects on neonatal rat cardiomyocytes [54], while others indicated that morphine enhances apoptosis in the kidneys and livers of mice [55].

Research in rats suggests that the depletion of antioxidant enzyme activity associated with drug abuse primarily results from oxidative stress [29]. Oxidative stress is a byproduct of lipid peroxidation [56]. It is well-established that morphine induces oxidative stress by generating ROS, which initiate and exacerbate oxidative damage, including lipid peroxidation [57]. MDA, a metabolite of lipid peroxidation, results from the reaction between superoxide anions (O_2_^–^) and polyunsaturated fatty acids [56]. In this study, MDA levels were significantly elevated in rats administered a low dose of morphine compared to both the control group and rats treated with medium and high doses, suggesting an atypical dose-response pattern likely resulting from morphine-induced lipid peroxidation. Furthermore, these findings corroborate those of Zhang *et al.* [58], who reported that morphine elevates oxidative stress markers, such as MDA and 8-hydroxy-2’-deoxyguanosine (8-OHdG), while concurrently depleting the activities of glutathione peroxidase (GPx), SOD, and CAT in the rat liver.

Reduced GSH levels in female rats administered all three drug doses were notably higher than in the control group. Karajibani *et al.* [56] demonstrated that ROS production and levels of total antioxidant capacity (TAC), GSH, vitamins C, E, and A, as well as the activities of CAT, SOD, and GPx, were increased in cases of opium dependence. SOD is a vital first-line antioxidant enzyme that detoxifies superoxide radicals (O_2_·^−^) into hydrogen peroxide (H2O2). This H2 O2 is subsequently converted into water by GPx and CAT. Together, these enzymes provide essential protection against free radical damage within cells.

A previous study by Ismail *et al.* [59] demonstrated that opium reduces GSH concentration in hepatic mitochondria, induces SOD activation, and diminishes GPx and CAT activities. However, in the current study, the SOD and CAT activities in rats treated with medium and high doses of morphine were significantly elevated compared to both the control group and the group receiving the low dose of the drug. This suggests an enhanced adaptive response to oxidative stress, characterized by the upregulation of CAT and SOD activities in these rats.

Morphine induces elevated nitric oxide (NO) production by modulating intracellular calcium concentrations. This process involves the activation of calcium/calmodulin-dependent NO synthase (NOS) and the upregulation of NO expression via naloxone-sensitive opioid receptors [60]. NO levels were significantly elevated in both low- and high-dose morphine-treated male rats compared to controls. Similarly, female rats receiving the low dose exhibited a significant increase in NO production. These findings suggest that morphine may exacerbate inflammatory responses, potentially by upregulating the expression of Toll-like receptors (TLRs) within hepatic tissue. These results align with those of Chehrei *et al.* [61], who demonstrated that morphine administration elevates nitric oxide levels in rat testes by enhancing the gene expression of TLRs. Moreover, Pajohanfar *et al.* [62] confirmed that morphine exposure in rats elevated levels of both Toll-like receptor 4 (TLR4) and inducible nitric oxide synthase (iNOS) in the brains of mice.

## Conclusion

This study concludes that the livers of female rats are more susceptible to the adverse effects of morphine than those of males. This is evidenced by altered liver enzymes and the presence of widened sinusoids observed in the histology of female rat livers. Rats administered a low dose of morphine exhibited significant changes in liver enzyme transaminases, as well as inflammatory and apoptotic markers, compared to those given medium and high doses of the drug. Further research should investigate the differential hepatic expression of CYP450 isoforms and the expression of drug efflux pumps on the cell membranes of male and female rat liver tissue, alongside biomarkers of DNA damage. Additionally, measuring progesterone and estrogen levels and assessing the affinity of their binding to hepatic receptors is warranted to elucidate the role of these sex hormones in the observed sex-specific differences in the liver’s response to morphine treatment. Employing polymerase chain reaction (PCR) and immunoblot analysis are suitable methodologies for these molecular assessments.

## Data Availability

The datasets generated and analyzed during the current study are available from the corresponding author upon request.
